# Searching for Scientific Explanations for the Uses of Spanish Folk Medicine: A Review on the Case of Mullein (Verbascum, Scrophulariaceae)

**DOI:** 10.3390/biology10070618

**Published:** 2021-07-02

**Authors:** José Blanco-Salas, María P. Hortigón-Vinagre, Diana Morales-Jadán, Trinidad Ruiz-Téllez

**Affiliations:** 1 Department of Vegetal Biology, Ecology and Earth Science, Faculty of Sciences, University of Extremadura, 06006 Badajoz, Spain; truiz@unex.es; 2 Department of Biochemistry, Molecular Biology and Genetics, Faculty of Sciences, University of Extremadura, 06006 Badajoz, Spain; 3 One Health Research Group, Universidad de las Américas, Campus Queri, Quito 170513, Ecuador; diana.moralesj91@gmail.com

**Keywords:** Verbascum, traditional knowledge, validation, flavonoid, terpene, inflammatory

## Abstract

**Simple Summary:**

Mullein (*Verbascum* spp.) has been widely used in Spanish folk medicine to treat several pathologies, and these applications suggest the potential anti-inflammatory action of these plants. Based on the aforementioned, a deep bibliographic review of the chemical composition of the 10 species of Verbascum, catalogued by the Spanish Inventory of Traditional Knowledge related to Biodiversity, and virtual simulations using computer programs were used to demonstrate the molecular evidence supporting the use of these intuitive and traditional popular medicines.

**Abstract:**

*Verbascum* species (common mullein) have been widely used in Spanish folk medicine to treat pathologies related to the musculature, skeleton, and circulatory, digestive, and respiratory systems, as well as to treat infectious diseases and organ-sense illnesses. These applications support the potential anti-inflammatory action of Verbascum phytochemicals. Based on the aforementioned facts, and following a deep bibliographic review of the chemical composition of the 10 species of Verbascum catalogued by the Spanish Inventory of Traditional Knowledge related to Biodiversity, we look for scientific evidences to correlate the traditional medical uses with the chemical components of these plants. To support these findings, in silico simulations were performed to investigate molecular interactions between Verbascum phytochemicals and cellular components. Most of common mullein traditional uses could rely on the anti-inflammatory action of phytochemicals, such as quercetin, and it could explain the employment of these plants to treat a wide range of diseases mediated by inflammatory processes such as respiratory diseases, otitis, arthrosis, and rheumatism among others.

## 1. Introduction

The genus Verbascum (Scrophulariaceae, Lamiales) comprises more than 300 Eurasiatic species. It is the largest genus of the family, and its origin is the center of the Eastern Mediterranean Basin. In the Iberian Peninsula, it is represented by 26 species [1]. In Spain, they are popularly named “gordolobos” (in English, common mullein), and the Spanish Inventory of Traditional Knowledge related to Biodiversity [2] has catalogued 10 species which have been used to treat a wide range of pathologies. These are *Verbascum pulverulentum* Vill., *V. sinuatum* L., *V. thapsus* L., *V. boerhavii* L., *V. creticum* (L.) Cav., *V. dentifolium* Delile, *V. giganteum* Willk., *V. lychnitis* L., *V. rotundifolium* Ten., and *V. virgatum* Stokes in With.

In order to realize the potential pharmacological application of these species, we must perform a deep analysis of their chemical compositions as a starting point to understand which phytochemicals could exert the medical actions described in the traditional knowledge. The chemical components of *Verbascum* spp., and the biological actions attributed to these phytochemicals, can be found in the literature [3,4,5,6,7,8,9,10,11,12,13,14,15,16,17], with the correlation between the phytochemicals’ bioactivity and their traditional uses being a key point to validate their traditional ethnobotanical uses.

The aforementioned bibliographic prospection could be complemented by in silico approaches to demonstrate the phytochemicals’ affinities using molecular targets. The combination of bibliographic research and computer programming could provide a strong tool to approach the botanical bioactive compounds existing in *Verbascum* spp. with the medical uses collected by folk knowledge.

The objective of this work is to analyze the affinities of phytochemicals from *Verbascum* spp. for mammalian molecular targets to perform a comprehensive scientific validation of its medical uses. This work could support further experimental studies on *Verbascum* spp. extracts and their phytochemicals as therapeutic agents, making the experimental approach easier and eventually contributing to reducing the number of animals employed in pre-clinical testing [18,19].

## 2. Materials and Methods

### 2.1. Ethnobotanical Uses and Chemical Composition of Verbascum Used in Spanish Folk Medicine

We first carried out a bibliographic search, looking at the applications recorded by the Spanish Inventory of Traditional Knowledge related to Biodiversity [2], for the 10 *Verbascum* spp. catalogued in the Iberian Peninsula. We summarized them in a table, grouped by diseases and physiological systems.

Afterwards, we performed a bibliographic review of the chemical composition of the 10 Verbascum species. We used the databases Scopus, Dialnet, Medline, PubMed, ScienceDirect, Google Patents, Google Scholar, and Wiley Online. The employed keywords were: “*Verbascum sinuatum*”, “*Verbascum thapsus*”, “*Verbascum boerhavii*”, “*Verbascum creticum*”, “*Verbascum dentifolium*”, “*Verbascum giganteum*”, “*Verbascum lychnitis*”, “*Verbascum rotundifolium*”, “*Verbascum virgatum*” and/or “activity”, “chemical composition”, “pharmacology”, and “medicin*”.

The bibliographic results were managed using a Prisma 2009 Flow Diagram Methodology [20]. A final summary was obtained. It contains the metabolites that had been identified in the aforementioned Verbascum species throughout the published literature and can be consulted in Appendix A (Table A1).

The chemical structures of these metabolites (83 molecules of Table A1) were retrieved from PubChem [21]. This is a database of chemical compounds maintained by the National Centre for Biotechnology Information (NCBI), a branch of the National Library of Medicine of the National Institute of Health (NIH). Structures were drawn and edited using ChemDraw Professional 17.0 (Perkin Elmer, Waltham, MA, USA) and/or Marvin Sketch 19.15 (ChemAxon, Budapets, Hungary). Finally, the respective SMILES codes were also compiled in the abovementioned Table A1 because they are essential to perform the in silico modelling planned for the next stage.

### 2.2. In Silico Modelling of Verbascum spp. Chemical Constituents’ Affinities by Human Molecular Targets

To obtain a virtual prediction of the probable molecular targets of the Verbascum metabolites listed in Table A1, we used the free Software SwissTargetPrediction (STP) [22]. This program allows one to estimate the most probable macromolecular targets of any small molecule assumed to be a bioactive metabolite. The prediction is founded on a combination of 2D and 3D similarity with a library of 370,000 known actives from more than 3000 proteins from 3 species. We focused our predictions on *Homo sapiens* targets. When a metabolite molecule SMILES code is uploaded to the SwissTargetPrediction Website, a document is obtained, which contains a list where proteins are ranked according to the probability of the protein being a target of the query molecule (phytocompounds). Probabilities of ≥0.65 are considered to be significant in the metabolite–protein interaction [22].

We uploaded each of the Verbascum metabolites to the SwissTargetPrediction System; the significant results are summarized in a table available in Appendix B (Table A2). It corresponds to the list of 20 metabolites which showed a significant level of affinity for different targets. The results of Table A2 were analyzed and presented as a frequency histogram figure, structured from the perspective of the STP Target Classes. 

The SwissTargetPrediction Program runs with a database system where the proteins included are linked to its own Class Target Classification System.

In summary, the total number of Verbascum metabolites tested in silico was 83, and the metabolites that showed target affinities (finally, 20) were then analyzed, studied, and discussed.

### 2.3. Comparative Review of Ethnobotanical Uses and Physiopatological Molecular Targets

The discussion consisted of making a qualitative comparison between the traditional use and biological activity of the components. The latter was considered in the published experimental results, which are accessible through bibliographic databases, and the in silico protein affinity tests performed using the aforementioned SwissTargetPrediction Program.

## 3. Results

### 3.1. Ethnobotanical Uses and Chemical Composition

The use of *Verbascum* spp. in Spanish traditional medicine includes a wide range of formulations to treat disorders affecting a wide range of systems such as the circulatory, digestive, and respiratory systems, as well as skin diseases, sense organ illnesses, and infectious and parasitic diseases. The main applications collected by the Spanish Inventory of Traditional Knowledge related to Biodiversity [23] for the 10 *Verbascum* spp. catalogued in the Iberian Peninsula are summarized in Table 1, in which we have also included data on the method of administration.

#### 3.1.1. Circulatory System Diseases

Among the circulatory system applications, the anti-hemorrhoidal use of *Verbascum* spp. is the best established, as it has been reported for 7 out of 10 Iberian species. Topical application is the most common posology; it can be accomplished by sitz bath, with the liquid resulting from plant decoction [24,25,26,27,28,29], or by rubbing the mash or boiled plant onto the affected area [24,30,31,32,33,34,35,36,37,38,39]. Rubbing with hairy leaves has also been reported [40,41,42].

#### 3.1.2. Digestive Apparatus

Digestive system illnesses, in many cases, include conditions caused by an inflammatory process (tooth pain, gumboils, liver and gastric inflammation). Moreover, these species have also been used for their digestive properties and to treat gallstones, diarrhea, and constipation. Again, the liquid resulting after boiling to decoct the plant is the most common posology, together with plant infusions, which are commonly drunk to obtain healing benefits [28,31,36,38,42,43,44,45,46,47,48,49]. Nevertheless, these species can also be used in mouthwashes to treat teeth pain and gumboils [36,38,50,51], or as enemas for constipation, pediatric gut swelling, and indigestion [25]. The topic application of poultices or leaves (boiled or raw) is also used to treat abdominal pain, commonly attributed to liver or gut inflammation or diarrhea [25,28,33,34,35,52,53].

#### 3.1.3. Respiratory Diseases

The most common way to use *Verbascum* spp., to relieve respiratory system conditions, such as hoarseness, tonsillitis, cold, cough, asthma, or bronchitis, is through the ingestion of a wide variety of preparations (infusions, macerations, syrup) made with common mullein alone or mixed with other plants (mint, rosemary, mallow, hawthorn flower, coltsfoot, thymus and pine leaves, among others) or culinary ingredients (honey and sugar) [24,25,28,30,31,33,35,36,38,39,40,43,45,46,49,51,54,55,56,57,58,59,60,61,62,63,64,65,66,67,68]. The ability of *V. thapsus* extracts to inhibit the growth of bacteria involved in respiratory infections has been proved using antibacterial assays, with the aqueous extracts being the most efficient [69].

#### 3.1.4. Musculature and Skeleton

Regarding the employment of *Verbascum* spp. to treat and relieve conditions affecting the musculature and skeleton, the healing properties attributed to common mullein could rely on its anti-inflammatory action, since most of the conditions treated share a strong inflammatory component (rheumatism, arthritis, swelling, contusions, and broken bones). The formulas employed include fresh, mashed, boiled, or infused plants, and the means of application is topical [25,26,29,30,33,35,43,52,67,70,71].

#### 3.1.5. Skin and Sense Organs

A wide range of skin conditions are treated with *Verbascum* spp., including eczema, exanthema, cysts and zits, insect bites, and nail infections, as well as different types of wounds. The topical application of the liquid, resulting from boiling, infusing, or macerating the plant, is the most common posology [24,25,26,28,29,31,33,35,36,39,41,42,45,46,47,48,52,54,57,60,61,68,72,73,74,75,76,77,78,79]. The species’ employment for chilblain relief is another common use (5 out of 10 *Verbascum* spp.). The most common means of application is rubbing the liquid, resulting from decoction [25,27,38,39,43,80,81,82], which, in Alicante, is carried out in milk instead of water [83]. In Caceres, a lead poultice is applied on the affected area [41].

A liniment made from mullein flowers, boiled or macerated in olive oil, is a common means for treating earache in different parts of Spain (Cataluña, Baleares, and Navarra) [24,25,35,66]. Conjunctivitis is another condition treated with common mullein [24].

#### 3.1.6. Other Uses

Finally, another interesting application of *Verbascum* spp. is the treatment of infectious and parasitic diseases, such as diphtheria, helminthiasis, tuberculosis, typhus, and mange [25,28,35,62,68,77]. Despite the lack of experimental results showing the anti-mycobacterial action of Verbascum extracts, the British folk knowledge also point to the ability of common mullein to treat tuberculosis. Besides it, the nomenclature and local names of this genus are tightly connected with diseases caused by mycobacteria [84].

#### 3.1.7. Chemical Composition

**Figure 1:**
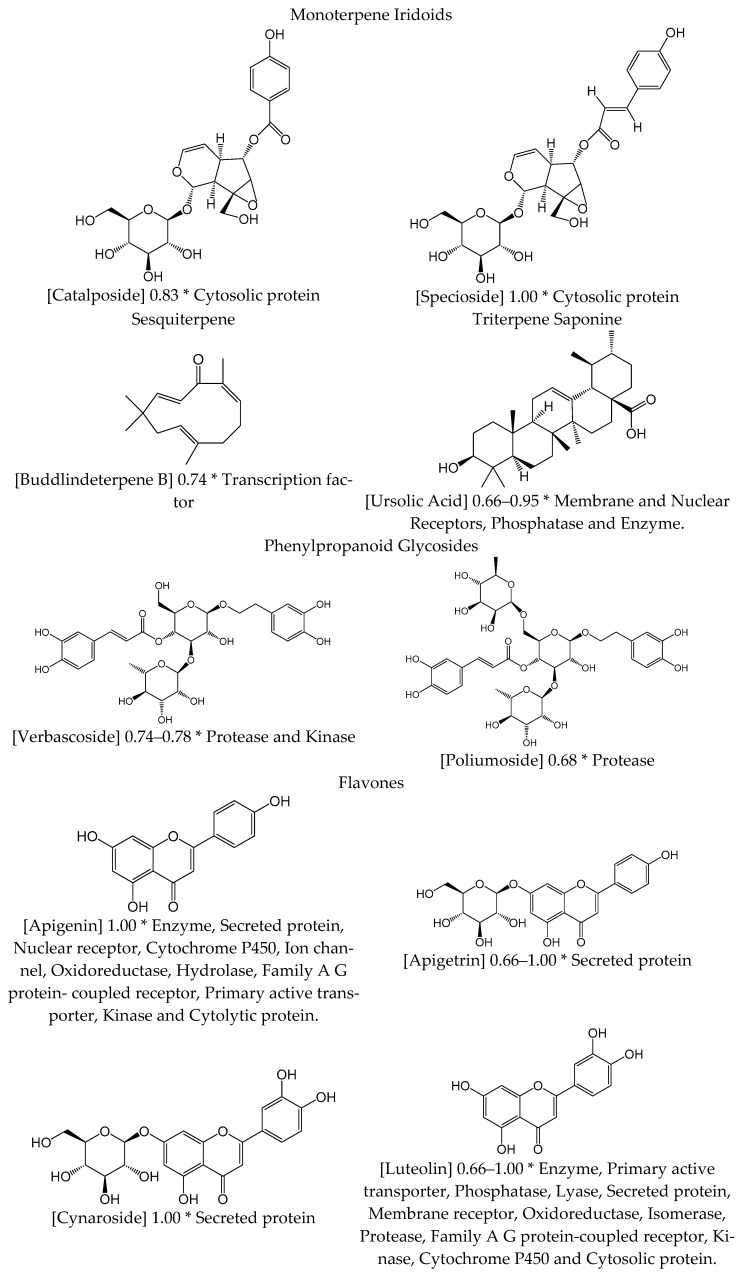

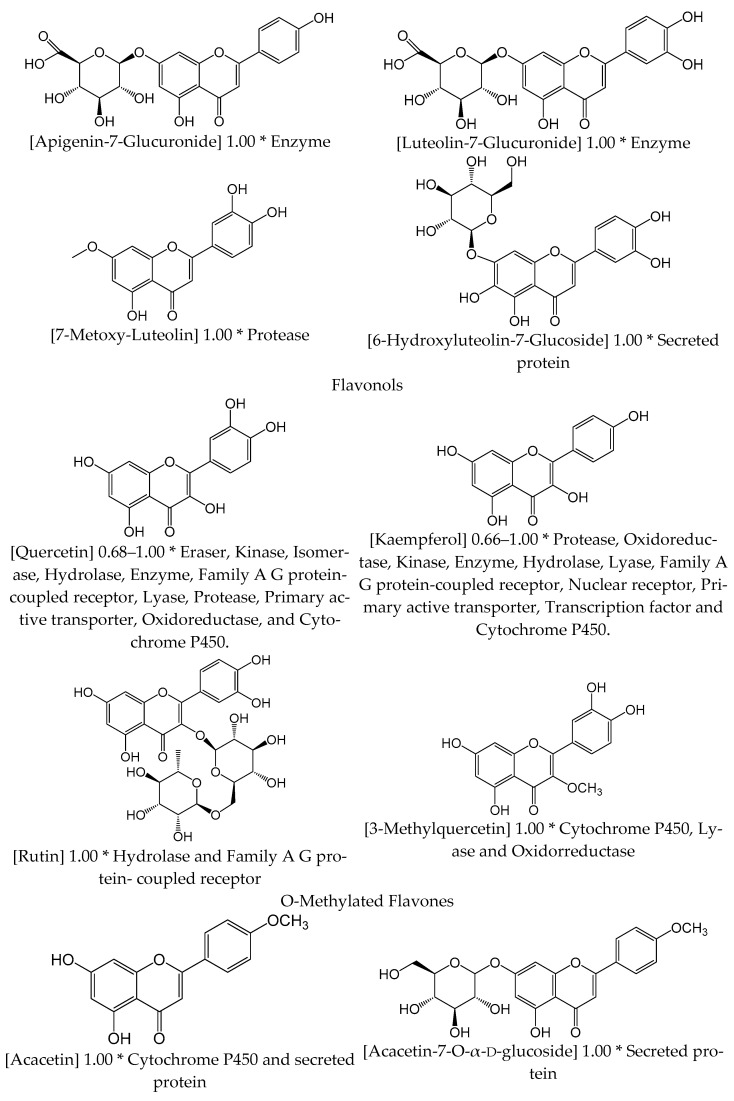
Chemical structures of Verbascum components with probability values and target class according to the SwissTargetPrediction classification. * Probability—target class.

Spanish *Verbascum* spp. phytocompounds include two main classes: terpenes and flavonoids (see Table A1 and Figure 1). The best characterized species are *V. thapsus* [3,4,6,9,15,16,17], *V. sinuatum* [10,11,12,13], and *V. lychnitis* [5,7,14].

Monoterpene iridoids, sesquiterpenes, triterpene saponins, and phenyl propanoids are isoprene derivatives. Monoterpene iridoids are 10 C terpenes with a cyclopentanopyran cycle. Catalposide and specioside are metabolites belonging to this group. Their chemical structures are very similar, though differing in the way the phenol group is inserted, with specioside being more hydrophobic. Sesquiterpenes are 15 C terpenes, such as buddlindeterpene B. Triterpene saponins (vg. ursolic acid) are 30 C terpenes that reduce the surface tension, easing the mix of lipophilic and hydrophilic phases from liquid substances. Phenilpropanoid alcohols are glycosidic molecules, such as verbascoside and poliumoside.

Flavonoids share a flavonic nucleus (2-phenylbenzopyrane). They have been classified into three subgroups: flavonols, flavones, and O-methylated flavones. Flavones are pheny1-4 benzopyranones, flavonols are 3-hidroxyflavones, and O-metilated flavones have a methyl radical in the 3-hydroxilated part of the main pheny1-4-benzopyranone nucleus. The flavonoid components of Table A1 have a common structure of chromone (1-4 benzopyranone); are characterized by main functional groups such as hydroxyl, and carbonyl; have a conjugated double bond. They are soluble in water and ethanol, and they have oxygen bases varying from moderate to strong.

Some of these components have a powerful physiological activity, which has been shown in several experimental works [85,86,87]. This activity, usually with a narrow therapeutic margin (little difference between the minimum active concentration and the maximum tolerated concentration), has attracted interest in its associated biochemical processes.

### 3.2. In Silico Modelling of Verbascum spp. Chemical Constituents’ Affinities by Human Molecular Targets

The review resulted in a library of 83 molecular structures identified in Verbascum. (Table A1). The application of the SwissTargetPrediction program yielded a final score of 20 molecules with ligand–target interactions with a probability of ≥0.65; thus, these were selected, and the rest were discarded. They are summarized in Table 2 and additional data are available in Table A2 (Appendix B).

The chemical structures of the 20 components are plotted in Figure 1, together with the probability values obtained by in silico modelling and target class, according to the SwissTargetPrediction classification.

**Figure 2:**
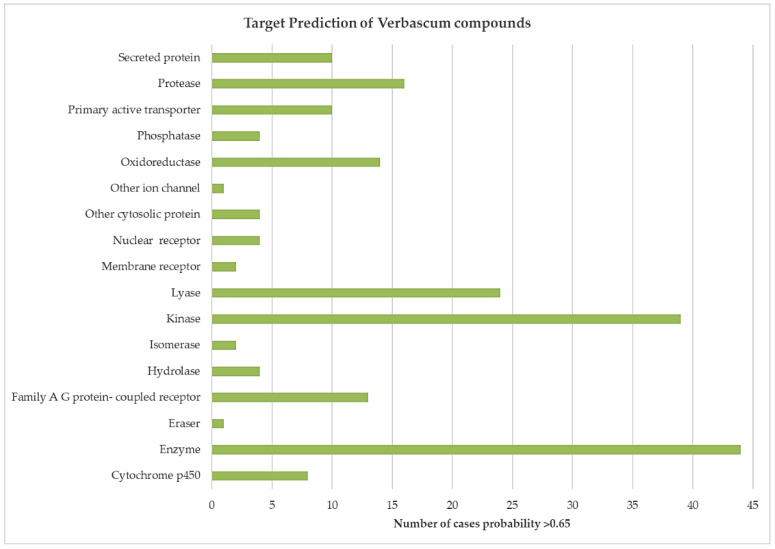
Quantification of cases where the Verbascum molecule–human target affinity is significant in the different classes of targets according to the SwissTargetPrediction classification system.

Figure 2 shows the quantification of cases where the probability is greater than 0.65, in relation to the target class established by SwissTargetPrediction, and shown in Table A2. It is necessary to emphasize the great affinity for the classes “enzymes” (44 cases), “kinases” (39 cases), and “lyases” (24 cases).

According to the data in Table A2, iridoids (catalposide, specioside) show affinity for the cytosolic protein HSP90AA1 (heat shock protein HSP90-α). The sesquiterpene, buddlindeterpene B, shows affinity for the transcription factors GLI1 and GLI2 (glioma-associated oncogen, which are zinc finger proteins). Ursolic acid, a triterpene saponin, mainly shows affinity for PTPN1 (protein-tyrosine phosphatase 1B) and other phosphatases (PTPN2 or T-cell protein-tyrosine phosphatase, P246666, or low molecular weight phosphotyrosine protein phosphatase), as well as the membrane receptor PTPRF (receptor-type tyrosine-protein phosphatase F), the nuclear receptor RORC (RAR-related orphan receptor γ), the DNA polymerase β (POLB), the aldo-ketoreductase 10 (AKR1B10), and the 11-beta hydroxysteroid dehydrogenase 1 (HSD11B1). The phenylpropanoid glycosides (verbascoside, poliumoside) show affinity for matrix metalloproteinases (MMP2, MMP12). The studied flavones (apigenin, apigenin-7-glucuronide, apigetrin, cynaroside, luteolin, luteolin-7-glucuronide, 6-hydroxyluteolin-7-glucoside, 7-methoxy-luteolin) show a wide profile of affinities, as summarized in Table A2. Among them are affinities for Cit P450, Glyoxalase 1 (GLO1), proinflammatory cytokine IL2, TNF-α secreted proteins, NADPH oxidase (NOX4), and arachinodate lipoxygenase (LOX), and the metalloproteinases (MMP 9 and 12) can be highlighted. The O-metilated flavones (acacetin, acacetin-7-O-α-d-glucoside) show affinity for cytochrome P450 (CYP1B1), interleukin-2 (IL2), and the Tumor Necrosis Factor (TNF-α).

## 4. Discussion

### 4.1. Anti-Inflammatory Action of Verbascum

The role of biological molecules, such as inteleukins (ILs), lipooxygenase (LOX), cyclooxygenase (COX), nuclear factor κB (NF-κB), vascular endothelial growth factor (VEGF), matrix matalloproteinases (MMPs), and tumor necrosis factor (TNF), among others, with the onset of inflammation is well known as well as the link between inflammation and chronic diseases [88]. Therefore, the study of phytochemicals, able to block the action of the aforementioned molecules, is key in the search of new drug candidates to treat chronic diseases and other pathologies with a high inflammatory component.

Most of medicinal applications of *Verbascum* spp. collected from the folk knowledge, have in common an array of inflammatory processes; therefore, understanding the anti-inflammatory molecular mechanisms displayed by Verbascum phytochemicals is essential in order to explain most of its healing properties.

The results generated by our affinities studies show the affinity of flavones (apigenin and luteolin) and flavonols (quercetin, 3′-methylquercetin and kaempferol) by arachinodate-lypoxygenases (LOX), a group of enzymes implicated in the synthesis of eicosanoids, such as leukotriens (LTs), which are molecules with an essential role in cell signaling, being also implicated in inflammation and disorders, such as asthma, skin diseases, rheumatoid arthritis, allergic rhinitis, inflammatory bowel, cardiovascular diseases, cancer, and osteoporosis [89,90,91,92,93]. It is well-known the anti-inflammatory role of polyphenolic compounds [94], in which flavones and flavonols are included. The ability of these compounds to interfere with enzymes implicated in the synthesis of eicosanoids, such as LOX, is one of the molecular mechanisms underlying their anti-inflammatory properties, and the ability of quercetin and lutein to suppress LOX product synthesis has been scientifically proven [90]. Despite our in silico approach cannot provide information about the molecular dynamic of phytochemical-target interaction, the affinity of flavones (apigenin and luteolin) and flavonols (quercetin, 3′-methylquercetin and kaempherol) for LOX, obtained by our in silico approach, is consistent with the scientific results found in the literature, in which the ability of quercetin and luteolin to suppress the formation of LOX products implicated in inflammation, such as LTs, is well demonstrated [90].

The polyphenolic compounds listed in Figure 1 shared a cathechol partial structure, which could be responsible for uncoupling the catalytic cycle of LOX, due to its iron chelating and antioxidant properties [90].

Another interesting result obtained from our in silico studies has shown the affinity of luteolin, quercetin, and kaempferol for interacting with NOX4 (NADPH oxidase-4), an enzyme implicated in the generation of superoxide anions and other downstream reactive oxygen species (ROS) [95]. For example, the protective role of luteolin against inflammation via the NOX4/ROS-NF-κB and MAPK pathways supports our findings and explains the anti-inflammatory action of mullein [96]. Compounds such as acacetin, apigetrin, and cynaroside have a high affinity to interact with the proinflammatory cytokines TNF-α and IL-2, which could also be related to their anti-inflammatory effects. In 2017, a paper from Hu et al. [97] demonstrated the anti-inflammatory effect of the flowers of Chuju (a medical cultivar of *Chrysanthemum morifolim* Ramat), which contain apigetrin and acacetin in their chemical composition [97]. A work of Zhao et al. (2014) [98] showed the ability of acacetin to block T-cell proliferation and IL-2 secretion, both essential to induce the inflammatory response underlying diseases such as rheumatoid arthritis and psoriasis [98]. The anti-inflammatory bioactivity of apigetrin has also been reported in an animal model of acute otitis media [99], which is a traditional use of Verbascum widely reported throughout the Iberian peninsula. Eventually, the anti-inflammatory effect of cynaroside has been demonstrated in a model of human periodontal ligament (hPDL) cells, a cell type essential in the maintenance of the periodontal tissues homeostasis, in which cynaroside has the ability to decrease the expression of pro-inflammatory cytokines, such as TNF-α, induced by LPS treatment [100].

Eventually, the in silico result, showing affinity between ursolic acid and the retinoic acid-related orphan receptor gamma (RORγ), a transcription factor essential for T helper cells differentiation, supported by experimental result showing an effective and selective inhibitory effect of this phytochemical over RORγ, could also explain the anti-inflammatory properties attributed to Verbascum spp [101].

### 4.2. Circulatory System Diseases

The most remarkable uses in this section are those related to circulation. The applications of these species against hemorrhoids and varicose veins are related to their local expansion processes in the peripheral circulation. This healing action can be explained by the presence of flavonoids, whose antioxidant and vasodilatory activities are associated with their protective cardiovascular action, widely referred to in the literature [102]. These compounds are common in aqueous extracts from the plants [103], so their presence is expected in many of the preparations recorded in Spanish traditional medicine and listed in Table 1. It has been reported that they are mainly used after being boiled and are then applied externally. The pathologies previously mentioned have also a local inflammatory component, therefore, the anti-inflammatory activity of common mullein, discussed in the previous section, could also underlie this group of healing remedies [93]. 

The antihypertensive use of *Verbascum* spp. reported in Table 1 could rely on the interaction of Verbascum phytocompounds with the α-adrenergic receptors implicated in peripheral vascular resistance walls. On the one hand, the α-adrenergic antagonist activity of flavonoids could explain Verbascum’s antihypertensive action [104]. On the other hand, the affinity of rutin to interact with the α2-adrenoreceptors obtained in our in silico assays, and its anti-hypertensive action reported in the literature [105], could contribute to the antihypertensive action of Verbascum reported from folk knowledge [106].

### 4.3. Digestive Apparatus

The digestive process begins with activity in the oral cavity, chewing, salivation, and swallowing. Therefore, oral health is essential for proper digestion. The employment of infusions and decoctions, of these plants by Spanish folk medicine, to treat tooth pain and gumboil could be related to the anti-inflammatory activity discussed above. The anti-inflammatory effect of common mullein could rely on the anti-inflammatory action of its phytochemical cynaroside which has been demonstrated to confer protection against the inflammation underlying the periodontitis [100].

Other applications include for digestive problems, gastric ulcer, or inflammations in different parts of the digestive system (stomach, liver, gallbladder), for which there are treatments described in the traditional Spanish uses of the plant (Table 1). One study indicates the protective effect of ursolic acid against hepatotoxicity in mice [107]. 

In addition, some of these proteins are specifically related to the physiology of the gastro-intestinal tract. Salivary amylases help to break down food into its molecular components. Parietal cells in the stomach release various acids, pepsins, and enzymes, including gastric amylase, to achieve partial digestion and obtain chemo (semi-fluid and semi-digested mass). Acids also neutralize salivary amylase, favoring gastric intervention. After about an hour, the chimo is pushed into the duodenum, where acidity acquired in the stomach stimulates the release of the hormone secretine. The pancreas then releases hormones, bicarbonate, bile, and numerous pancreatic enzymes, such as lipases (P04054), and those of the lipidic metabolism, such as aldoreductases and most of the ones consigned in the “Enzyme” file of Table A2. These are related to glucose conversion in NADPH-dependent sorbitol, the first step in the poliol pathway of glucose metabolism [108]. Afterwards, thanks to bicarbonate, the acidity of the chimo is changed into an alkaline form, allowing the better degradation of food and also creating a hostile environment for bacteria that survived the passage to the stomach. This process can be carried out effectively and smoothly if the enzyme system is healthy; otherwise, careful supplementation is required [109].

More difficult to validate, however, is the use related to defecation processes. These species have been used as both astringents and laxatives, and the only possible explanation for the traditional use of these plants is that in the first case, diarrhea (for which infusions are taken) has some infectious origin and causes inflammation. In the second case, where enemas are used because of the evacuating effect achieved by the mechanical action of water, this is favored by the presence of triterpene saponins, which have the ability to produce soapy solutions.

### 4.4. Respiratory Diseases

Respiratory tract pathologies treated with mullein have different etiologies (hoarseness, tonsilitis, colds, coughs, asthma, bronchitis, and even hemoptysis) and treatments, but all have a common feature: the development of inflammatory processes. Besides this, in many cases, fever and cough are displayed. The relief properties of mullein could be explained by its antitussive and expectorant activities, which could be justified by the presence of mucilages in these species [110] which exert demulcent activity [111].

Ursolic acid is one of the most promising substances of biological origin for antimicrobial therapy. It has been identified as a phytochemical inhibitor of the main protease of COVID-19 using molecular modelling approaches [112,113,114]. Other potential phytochemicals of Verbascum spp., which could be useful to treat COVID-19, are the flavonoids apigenin, luteolin, and quercetin, which have been shown to be replication inhibitors of other coronaviruses [115].

Since, in severe COVID-19 patients, an elevation of pro-inflammatory cytokines occurs, also known as “cytokine storm”, that is responsible of deteriorating their health conditions, the search of drugs able block target this “cytokine storm” and suppress the exacerbated inflammatory response is key in the treatment of the complications associated to the disease [116]. Our in silico results have evidenced affinity between mullein phytochemicals (Flavones and O-metilated flavones) and pro-inflammatory cytokines (IL-2 and TNF-α), molecules implicated in inflammatory processes related to the respiratory system and COVID-19 [117,118,119]. The previously validated anti-inflammatory activity of Verbascum components also supports the potential use of the extracts from the plants tackled in this review to achieve the desire anti-inflammatory action requested to prevent and treat COVID-19 acute clinical profile. The employment of natural compounds with immunosuppressant properties could be useful as adjuvants to ameliorate the inflammatory process triggered by the out-of-control immune response which could be fatal for the patient, even causing death [120].

Our hypothesis suggesting the employment of Verbascum flavonoids as promising COVID-19 treatment is extensively supported by the existing literature which includes a large number of works using in silico and in vitro approaches which demonstrate the ability of flavonoids to interfere with the viral infection or to prevent/ameliorate the COVID-19 disease effects. Among SARS-CoV2 targets blocked by flavonoids 3CL^pro^ (the protease responsible of processing the two polyproteins firstly translated after viral entry) can be highlighted due to its pivotal role in the initiation and progression of the viral cycle and the lack of its human homologue. Apigenin, luteolin, kaempferol, and quercetin are able to inhibit the proteolytic activity of 3CL^pro^, quercetin being the most effective. The ability of these phytochemicals to interact with 3Cl^pro^ could be due to the ability of the two phenyl groups of flavonoids to interact with the protease substrate binding pocket [121]. Another target is the RNA-dependent RNA polymerase (RdRp) responsible or virus genome replication. The RdRp activity, and therefore the viral replication, is affected by high Zn^2+^ levels and quercetin can act as Zn^2+^ ionophore facilitating the influx of Zn^2+^ into the cell [122]. The last molecular target to deal with SARS-CoV-2 infection is to block the interaction between the SARS-CoV-2 Viral Spike Protein (S) and its cellular receptor, the Angiotensin Converting Enzyme-2 (ACE2) protein, responsible of viral entry. In silico experiments have shown the capacity of two flavonoids (quercetin and luteolin) to block this process [123,124].

A recent review work has studied the potential action mechanisms of Chinese Traditional Medicines to treat COVID-19 by targeting key proteins for the initiation and progression of the disease (ACE 2 and 3CL^pro^) or inhibiting inflammatory mediators. The formulas tackled by this review shared components presented in Verbascum spp. such as luteolin, kaempferol and quercetin [125]. 

The main challenge found in the use of flavonoids, such as quercetin, with a widely supported antiviral action is the poor oral bioavailability due to its reduced absorption and biotransformation during digestion [126,127]. This issue can be tackled through alternative administration ways, such as nasal spray [128] or phytosomes [129].

### 4.5. Musculature and Skeleton

The use of analgesic, anti-inflammatory, and/or antipyretic drugs is very common in treating a wide range of medical conditions in current clinical pharmacology. Traditional medicine has also used many plants with identical purposes, such as the *Verbascum* spp. studied here. The applications listed in Table 1 extracted from the Spanish National Inventory include a wide spectrum of remedies to treat osteoarthritis, rheumatism, hand crack, kneeache, gout footache, contusions, and even broken bones, all of them characterized by the onset of inflammation and pain. The main aspects considered in the preceding paragraphs have already been discussed within inflammation section.

Pain has been defined by the IASP (International Association for the Study of Pain) as an unpleasant sensory and emotional experience associated with or resembling that associated with actual or potential tissue damage [130]. The phenomenon is a multidimensional entity and nuanced elements of pain are not easy to apprehend when pain is measured with the standard qualitative metrics [130]. From a biochemical and molecular biology point of view, the relationship of certain proteins with painful effects is well known [131], although the potential utility of proteomics to investigate pain management has just started to be considered. Cytochrome P450 [132], gyoxalase I [133], myeloperoxidase [134], and kinases [135] are proteins involved in the physiopathology of pain. Table A2 summarizes how the in silico study points to the great affinity of phytocompounds of these vegetables—particularly quercetin, kaempferol, apigenin, and luteolin—with these proteins.

Osteoarthritis, one of the illnesses treated with common mullein by Spanish traditional medicine, is characterized by the degradation of cartilage, inflammation, and osteophyte formation in joints. Metalloproteinases are directly related to the onset of this medical condition due to their ability to proteolyze the extracellular matrix [136]. The affinity of some Verbascum phytochemicals (verbascoside, poliumoside, luteolin, quercetin, and kaempferol) for metalloproteinases could explain the traditional employment of mullein in osteoarthritis treatments. This notion is supported by a recent work which suggests the employment of verbascoside to treat osteoarthritis [136]. The employment of an ethanolic extract of Moussonia deppeana (high verbascoside content) shows an anti-edematous action in an experimental model of arthritis [137]. The ability of quercetin to reduce the severity of rheumatoid arthritis has also been demonstrated in vivo [138]. Another molecular mechanism, implicated in rheumatoid arthritis, is the invasion of fibroblast-like synoviocytes (FLS), which is responsible for cartilage destruction. Again, the metalloproteinases are involved in FLS invasion and kaempferol is able to reduce FLS migration and invasion both in vitro and in vivo [139].

A similar reasoning can be found regarding fever. Antithermic action is related to TNF-α secreted proteins [140] (P01375, Table A2), which have shown an in silico affinity with Verbascum flavones (6-hydroxyluteolin-7-glucoside, apigetrin, and cynaroside) and O-metilated flavones, such as acacetin-7-O-α-d-glucoside.

### 4.6. Skin and Sense Organs

The topical dermatological use of various extracts (infusion, boiling, maceration) from these plants for the treatment of occasional or repetitive local eruptions (cysts, zits, eczemas, exanthemas), accidental or more serious conditions (wounds, ulcers, burns, bites), and even eye or ear inflammations are justified by their anti-inflammatory power reported throughout this manuscript.

The employment of common mullein to treat otitis could be explained by the presence of apigetrin in its chemical composition. We have shown the high affinity of apigetrin for TNF-α and IL-2 (P01375 and P60558, respectively), both belonging to the cytokine family and implicated in inflammatory processes. This hypothesis is supported by a recent work which demonstrates the healing effect of apigetrin in otitis media due to its ability to suppress inflammation and oxidative stress. Treatment with apigetrin reduces mucosa thickness, inhibits the inflammatory response by downregulating neutrophils and macrophages, and reduces ROS generation, eventually alleviating otitis [99].

### 4.7. Other Uses

Other popular uses, such as in the treatment of infectious diseases and parasitosis (diphtheria, helminthiasis, tuberculosis, typhus, and mange), require a direct validation that is difficult to explain with the data currently available. Indirectly, all the anti-inflammatory actions discussed throughout this work need to be taken into consideration.

## 5. Conclusions

The use of Spanish Verbascum spp. is in traditional medicine as a healing plant related to various pathologies, most of them involving inflammatory processes, can be justified from a scientific point of view, based on the chemical composition of these plants and the biological activities tested in vitro or in vivo employing the isolated phytochemicals or the plant extract itself, which can be found through the large bibliographic databases surveyed. The bibliographic prospection is supported by a simple in silico approach to look for specific protein affinities, in order to conduct the aforesaid bibliographic search.

The popular and most common use of Verbascum spp. is linked to its anti-inflammatory properties, which could be explained by the presence of flavonoids such as luteolin, quercetin, apigenin, and kaempferol within chemical composition. The anti-inflammatory properties of these molecules are well validated in the literature. Our in silico study’s findings are in line with the experimental results found in the existing bibliography and have allowed us to select the phytochemicals with potential biological activities, among the preliminary list of compounds. This approach validates the employment of simple in silico studies aimed to obtain the molecule-target affinities as a useful tool to be employed before starting bibliographic or experimental works aimed to validate the biological activities of phytochemicals. This kind of studies have a pivotal role to underlie the search of potential pharmacological compounds to be used as drug candidates to treat a wide range of pathologies. In the case of the species studied, the activity of molecules such as the flavonoids (apigenin, apigetrin, cynaroside, luteolin, quercetin, kaempferol, rutin, acacetin), iridoids (catalposide, specioside), phenylpropanoids (verbascoside, poliumoside), sesquiterpenes (buddlindeterpene), and saponins (ursolic acid) could serve as inspiration for the design of improved drugs to treat a wide range of pathologies, including respiratory pathologies, which are of particular interest at the moment, in the context of the COVID 19 pandemic.

## Figures and Tables

**Table 1:** Traditional uses of Spanish Verbascum.

	Uses	*Vp*	*Vs*	*Vt*	*Vb*	*Vc*	*Vd*	*Vg*	*Vl*	*Vr*	*Vv*
Circulatory	Anti-hemorrhoidal	B/T	B/T	S/B/T	B/T			T	B	T	
	Leg treatment			B							
	Anti-hypertensive			I/B				I		B	
Digestive	Teeth pain, gumboil	B/T	B	B/T							T
	Digestive	I/B/T		B/T							
	Gastric ulcer/inflammation	B/T	I/B/T	B							
	Liver inflammation	I/B	T	I/B/T				I/B			
	Gallstone	I	I	I/B				I			
	Anti-diarrhoea	T	I	T							
	Constipation			B					E		
Respiratory	Hoarse, tonsillitis	B/T	I/T	I/B/T							
	Cold	B	I	I/B				I			B
	Cough, asthma, bronchitis, hemoptysis	B	I/B/M	I/B			I	I			B
Musculature & Skeleton	Anti-inflammatory (swelling)	B/T		I/B/T							
	Contusion, broken bones	I/T	T	I/B/T							
	Arthrosis, rheumatism		B/T	B/T				I		T	
Skin	Eczema, exanthema	B/T	B/T	T							
	Cysts and zits	T	T	I/B/T				T		T	
	Wounds, ulcers, burns	B/T	M/T	I/B/M/	T			T			
	Horsefly bite			M/T							
	Chilblain	B/T	B	B/T				B/T		B/T	
	Nail conditions			B/T							
Sense	Conjunctivitis	M	M	M							
	Otitis	B/M	M	B							
Infectious parasitic diseases	Diphtheria	T									
	Helminthiasis		B								
	Tuberculosis			I							
	Typhus			T							
	Mange			T							

(*Vp: V. pulverulentum; Vs: V. sinuatum; Vt: V. tapsus; Vb: V. boerhavii; Vc: V. creticum; Vd: V. dentifolium; Vg: V. giganteum; Vl: V. lychnitis; Vr: V. rotundifolium; Vv: V. virgatum).* Administration T: Topic; I: Infusion; B: Boiled; M: Maceration; E: Enem; S: Steam.

**Table 2:** Chemical constituents of Spanish Verbascum, which is used in folk medicine, with a ligand–target interaction probability of ≥0.65 calculated by the SwissTargetPrediction software.

Chemical Group	Component	Species
Monoterpene iridoid	Catalposide	*Vl*
	Specioside	*Vl*
Sesquiterpene	Buddlindeterpene B	*Vt*
Triterpene saponin	Ursolic acid	*Vt*, *Vl*
Phenypropanoid Glycosides	Verbascoside	*Vs*, *Vl*
	Poliumoside	*Vs*, *Vt*, *Vb*
Flavones	Apigenin	*Vt*
	Apigenin-7-glucuronide	*Vt*
	Apigetrin	*Vt*, *Vl*
	Cynaroside	*Vt*, *Vl*
	Luteolin	*Vt*, *Vl*
	Luteolin-7-glucuronide	*Vl*
	6-hydroxyluteolin-7-glucoside	*Vt*, *Vl*
	7-methoxy-luteolin	*Vl*
Flavonol	Quercetin	*Vt*, *Vl*
	3′-methylquercetin	*Vt*, *Vl*
	Kaempferol	*Vt*
	Rutin	*Vt*
O-metilated Flavone	Acacetin	*Vt*
	Acacetin-7-O-α-d-glucoside	*Vt*

*Vl = V. lychnitis, Vt = V. thapsus; Vs = V. sinuatum; Vb = V. boerhavii.*

## Data Availability

Not applicable.

## Abbreviations

*Vp*	*Verbascum pulverulentum*
*Vs*	*V. sinuatum*
*Vt*	*V. tapsus*
*Vb*	*V. boerhavii*
*Vc*	*V. creticum*
*Vd*	*V. dentifolium*
*Vg*	*V. giganteum*
*Vl*	*V. lychnitis*
*Vr*	*V. rotundifolium*
*Vv*	*V. virgatum*
SMILES	Simplified Molecular Input Line Entry Specification
IASP	International Association for the Study of Pain

## Appendix A

**Table A1:** Metabolites of Spanish Verbascum (*Vp: V. pulverulentum; Vs: V. sinuatum; Vt: V. thapsus; Vb: V. boerhavii; Vc: V. creticum; Vd: V. dentifolium; Vg: V. giganteum; Vl: V. lychnitis; Vr: V. rotundifolium; Vv: V. virgatum*) and SMILES code.

Metabolite		Species	Reference	SMILES Code
**Monoterpene Iridioids**				
1	Aucubin	*Vs*, *Vt*, *Vl*, *Vv*	[6,9,17]	C1=COC(C2C1C(C=C2CO)O)OC3C(C(C(C(O3)CO)O)O)O
2	6-O-β-d-glucopyranosyl aucubin	*Vs*	[9]	[H][C@@]23C=CO[C@@H](OC1[C@H](O)O[C@H](CO)[C@@H](O)[C@@H]1O)[C@]2([H])C(CO)=C[C@H]3O[C@H]4[C@H](O)O[C@H](CO)[C@@H](O)[C@@H]4O
3	Sinuatol	*Vs*	[9,11]	[H][C@@]34C=COC(O[C@H]2O[C@](CO)(O[C@@H]1O[C@@H](C)[C@H](O)[C@@H](O)[C@H]1O)[C@@H](O)[C@H](O)[C@H]2O)[C@]3([H])C(CO)=CC4
4	6-O-β-d-xylopyranosyl aucubin	*Vs*, *Vt*	[6,9,10,17]	[H][C@@]23C=CO[C@@H](O[C@H]1[C@H](O)O[C@H](CO)[C@@H](O)[C@@H]1O)[C@]2([H])C(CO)=C[C@H]3O[C@H]4[C@@H](O)O[C@H](CO)[C@@H]4O
5	6-O-α-l-sinuatosyl aucubin	*Vs*	[9,12]	[H][C@@]23C=CO[C@@H](O[C@H]1O[C@@H](CO)[C@H](O)[C@@H](O)[C@@H]1O)[C@]2([H])C(CO)=C[C@H]3OC5O[C@@H](CO)[C@H](O)[C@@H](OC4OCC(O)C(O)C4O)[C@@H]5O
6	Sinuatoside	*Vs*	[9,13]	OCC4=C[C@@H](OC2OC(CO)C(O)C(OC1OCC(O)C(O)C1O)C2O)C5C=CO[C@@H](OC3OC(CO)C(O)C(O)C3O)C45
7	Aucuboside	*Vs*, *Vl*	[9,13]	C1=COC(C2C1C(C=C2CO)O)OC3C(C(C(C(O3)CO)O)O)O
8	Catalpol	*Vs*, *Vt*, *Vl*	[6,9,17]	C1=COC(C2C1C(C3C2(O3)CO)O)OC4C(C(C(C(O4)CO)O)O)O
9	Isocatalpol	*Vt*, *Vl*	[6,9,17]	[H][C@@]24C=CO[C@@H](OC1OC(CO)C(O)[C@H](O)C1O)[C@]2([H])[C@@]3(CO)O[C@H]3[C@H]4O
10	Methylcatalpol	*Vt*, *Vl*	[6,9,17]	CO[C@@H]1[C@@H]2O[C@]2(CO)[C@H]2[C@H](O[C@@H]3O[C@H](CO)[C@@H](O)[C@H](O)[C@H]3O)OC=C[C@@H]12
11	6-O-α-l-rhamnopyranosylcatalpol	*Vt*	[6,9,17]	CC6O[C@H](O[C@H]3C2C=CO[C@@H](OC1OC(CO)C(O)[C@@H](O)[C@H]1O)C2[C@]4(O)O[C@H]34)C(COC(=O)C=Cc5ccccc5)C(O)[C@@H]6O
12	Saccatoside	*Vt*, *Vv*	[6,9,17]	C[C@H]1[C@@H]([C@H]([C@H]([C@@H](O1)O[C@H]2[C@@H]3C=CO[C@H]([C@@H]3[C@@]4([C@H]2O4)CO)O[C@H]5[C@@H]([C@H]([C@@H]([C@H](O5)CO)O)O)O)OC(=O)/C=C/C6=CC=C(C=C6)O)O)O
13	6-O-(3″-O-p-coumaroyl)-α-Lrhamnopyranosylcatalpol	*Vs*, *Vt*, *Vv*	[6,9,17]	C[C@@H]6O[C@@H](O[C@@H]3C2C=CO[C@H](O[C@@H]1OC(CO)[C@H](O)C(O)[C@@H]1O)C2[C@@]4(CO)O[C@@H]34)C(O)C(OC(=O)C=Cc5ccc(O)cc5)[C@@H]6O
14	6-O-(4″-O-p-coumaroyl)-α-Lrhamnopyranosylcatalpol	*Vt*	[6,9,17]	[H][C@@]24C=CO[C@@H](O[C@@H]1OC(CO)[C@H](O)[C@H](O)C1O)[C@]2([H])[C@@]3(CO)O[C@H]3[C@H]4O[C@H]5O[C@@H](C)C(O)C(O)C5OC(=O)C=Cc6ccc(O)cc6
15	6-O-(2″-O-(p-methoxy-trans-cinnamoyl)-α-l-rhamnopyranosylcatalpol	*Vt*	[6,9,17]	COc6ccc(C=CC(=O)OC1C(O)[C@H](O)[C@H](C)O[C@@H]1O[C@H]4C3C=CO[C@@H](O[C@H]2OC(CO)[C@@H](O)C(O)[C@H]2O)C3[C@]5(CO)O[C@H]45)cc6
16	6-O-(3″-O-(p-methoxy-trans-cinnamoyl)-α-l-rhamnopyranosylcatalpol	*Vt*	[6,9,17]	COc6ccc(C=CC(=O)OC5[C@H](O)[C@H](C)O[C@H](O[C@H]3C2C=CO[C@@H](O[C@H]1OC(CO)[C@@H](O)C(O)[C@H]1O)C2[C@]4(CO)O[C@H]34)C5O)cc6
17	Verbascoside A	*Vt*	[6,9,17]	C[C@H]1[C@@H]([C@H]([C@H]([C@@H](O1)O[C@H]2[C@@H]3C=CO[C@H]([C@@H]3[C@@]4([C@H]2O4)CO)O[C@H]5[C@@H]([C@H]([C@@H]([C@H](O5)CO)O)O)O)O)O)OC(=O)/C=C/C6=CC=C(C=C6)OC
18	6-O-[2″-O-(3,4-dihydroxy-trans-cinnamoyl)]-α-l-rhamnopyranosylcatalpol	*Vt*	[6,9,17]	C=C(C=Cc1ccc(O)c(O)c1)OC2C(O)[C@H](O)[C@H](C)O[C@@H]2O[C@H]5C4C=CO[C@@H](O[C@H]3OC(CO)[C@@H](O)C(O)[C@H]3O)C4[C@]6(CO)O[C@H]56
19	6-O-[4″-O-(3,4-dihydroxy-trans-cinnamoyl)]-α-l-rhamnopyranosylcatalpol	*Vt*	[6,9,17]	C=C(C=Cc1ccc(O)c(O)c1)O[C@@H]6[C@H](C)O[C@H](O[C@H]4C3C=CO[C@@H](O[C@H]2OC(CO)[C@@H](O)C(O)[C@H]2O)C3[C@]5(CO)O[C@H]45)C(O)C6O
20	6-O-[3″-O-(3,4-dimethoxy-trans-cinnamoyl)]-α-l-rhamnopyranosylcatalpol	*Vt*	[6,9,17]	C=C(C=Cc1ccc(OC)c(OC)c1)OC6[C@H](O)[C@H](C)O[C@H](O[C@H]4C3C=CO[C@@H](O[C@H]2OC(CO)[C@@H](O)C(O)[C@H]2O)C3[C@]5(CO)O[C@H]45)C6O
21	6-O-(2″-O-feruloyl)-α-l-rhamnopyranosylcatalpol	*Vt*	[6,9,17]	COc6cc(C=CC(=O)OC1C(O)[C@H](O)[C@H](C)O[C@@H]1O[C@H]4C3C=CO[C@@H](O[C@H]2OC(CO)[C@@H](O)C(O)[C@H]2O)C3[C@]5(CO)O[C@H]45)ccc6O
22	6-O-(4″-O-feruloyl)-α-l-rhamnopyranosylcatalpol	*Vt*	[6,9,17]	COc6cc(C=CC(=O)O[C@@H]5[C@H](C)O[C@H](O[C@H]3C2C=CO[C@@H](O[C@H]1OC(CO)[C@@H](O)C(O)[C@H]1O)C2[C@]4(CO)O[C@H]34)C(O)C5O)ccc6O
23	6-O-(2″-O-isoferuloyl)-α-l-rhamnopyranosylcatalpol	*Vt*	[6,9,17]	COc6ccc(C=CC(=O)OC1C(O)[C@H](O)[C@H](C)O[C@@H]1O[C@H]4C3C=CO[C@@H](O[C@H]2OC(CO)[C@@H](O)C(O)[C@H]2O)C3[C@]5(CO)O[C@H]45)cc6O
24	6-O-(3″-O-isoferuloyl)-α-l-rhamnopyranosylcatalpol	*Vt*	[6,9,17]	COc6ccc(C=CC(=O)OC5[C@H](O)[C@H](C)O[C@H](O[C@H]3C2C=CO[C@@H](O[C@H]1OC(CO)[C@@H](O)C(O)[C@H]1O)C2[C@]4(CO)O[C@H]34)C5O)cc6O
25	6-O-(4″-O-isoferuloyl)-α-l-rhamnopyranosylcatalpol	*Vt*	[6,9,17]	COc6ccc(C=CC(=O)O[C@@H]5[C@H](C)O[C@H](O[C@H]3C2C=CO[C@@H](O[C@H]1OC(CO)[C@@H](O)C(O)[C@H]1O)C2[C@]4(CO)O[C@H]34)C(O)C5O)cc6O
26	Pulverulentoside I	*Vp*, *Vs*, *Vt*	[6,9,17]	C=C(C=Cc1ccc(OC)cc1)OC6[C@@H](O[C@H]4C3C=CO[C@@H](O[C@H]2OC(CO)[C@@H](O)C(O)[C@H]2O)C3[C@]5(CO)O[C@H]45)O[C@@H](C)[C@@H](O)C6OOC(C)=O
27	6-O-(2″-O-p-methoxy-trans-cinnamoyl-4″-O-asetyl)-α-l-rhamnopyranosylcatalpol	*Vt*	[6,9,17]	COc6ccc(C=CC(=O)OC1C(O)[C@H](OC(C)=O)[C@H](C)O[C@@H]1O[C@H]4C3C=CO[C@@H](O[C@H]2OC(CO)[C@@H](O)C(O)[C@H]2O)C3[C@]5(CO)O[C@H]45)cc6
28	Pulverulentoside II	*Vp*	[9]	COc6ccc(C=CC(=O)OC5C(O)C(C)OC(OC3C2C=COC(OC1OC(CO)C(O)C(O)C1O)C2C4(CO)OC34)C5OC(C)=O)cc6O
29	Catalposide	*Vl*	[5,9]	C1=COC(C2C1C(C3C2(O3)CO)OC(=O)C4=CC=C(C=C4)O)OC5C(C(C(C(O5)CO)O)O)O
30	Specioside	*Vl*	[4,83]	C1C(C(=CC(=O)OC2=C1C=CC(=C2)OC3C(C(C(C(O3)CO)O)O)O)C4=CC=C(C=C4)O)O.C1C(C(=CC(=O)OC2=C1C=CC(=C2)O)C3=CC=C(C=C3)O)OC4C(C(C(C(O4)CO)O)O)O
31	Ajugol	*Vt*, *Vv*	[6,9,17]	CC1(CC(C2C1C(OC=C2)OC3C(C(C(C(O3)CO)O)O)O)O)O
32	6-O-benzoyl ajugol	*Vt*	[6,9,17]	[H][C@@]23C=CO[C@@H](O[C@@H]1O[C@H](CO)[C@@H](O)[C@H](O)[C@H]1O)[C@]2([H])[C@@](C)(O)C[C@H]3OC(=O)c4ccccc4
33	6-O-syringoyl ajugol	*Vt*	[6,9,17]	CC1(CC(C2C1C(OC=C2)OC3C(C(C(C(O3)CO)O)O)O)OC(=O)C4=CC(=C(C(=C4)OC)O)OC)O
34	6-O-vanilloyl ajugol	*Vt*	[6,9,17]	CC1(CC(C2C1C(OC=C2)OC3C(C(C(C(O3)CO)O)O)O)OC(=O)C4=CC(=C(C=C4)O)OC)O
35	Harpagide	*Vs*, *Vt*	[6,9,17]	CC1(CC(C2(C1C(OC=C2)OC3C(C(C(C(O3)CO)O)O)O)O)O)O
36	Harpagoside	*Vp*, *Vs*, *Vt*	[6,9,17]	CC1(CC(C2(C1C(OC=C2)OC3C(C(C(C(O3)CO)O)O)O)O)O)OC(=O)C=CC4=CC=CC=C4
37	Lychnitoside	*Vl*	[9]	OCC2=CO[C@@H](O[C@@H]1O[C@H](CO)[C@@H](O)[C@H](O)[C@H]1O)C3C=CCC23
38	Lateroside	*Vt*	[8]	[H][C@@]24C=CO[C@@H](O[C@@H]1O[C@H](CO)[C@@H](O)[C@H](O)[C@H]1O)[C@]2([H])[C@@](C)(OC(=O)C=Cc3ccccc3)C[C@H]4O
39	5-O-α-l-rhamnopyranosy (1α-3)-[α-d-glucuronopyranosyl (1α-6)]-α-d-glucopyranoside	*Vt*	[8]	CC8OC(OC1C(O)C(O)C(O)OC1OC2C(O)C(O)C(C(=O)O)OC2OC7CC[C@]6(C)C5CC=C4C3CC(C)(C)CC(O)[C@]3(C)CC[C@@]4(C)[C@]5(C)CCC6C7(C)CO)C(O)C(O)C8O
40	Ningpogenin	*Vt*	[8]	[H][C@]12C=C(CO)[C@@H](CO)[C@@]1([H])CC(=C)O2
41	10-deoxyeucommiol	*Vt*	[8]	CC1=C(CO)C(CCO)[C@@H](O)C1
42	Jioglutolide	*Vt*	[8]	C[C@@]1(C[C@H]([C@H]2[C@@H]1COC(=O)C2)O)O
43	6-β-hydroxy-2-oxabicyclo [4.3.0]Δ8-9-nonen-1-one	*Vt*	[8]	[H][C@@]12CCOC(=O)C1=C(C)C[C@H]2O
44	8-cinnamoylmyoporoside	*Vt*	[8]	C[C@@]1(C[C@H](C2[C@@H]1[C@@H](OC=C2)O[C@H]3[C@@H]([C@H]([C@@H]([C@H](O3)CO)O)O)O)O)OC(=O)/C=C/C4=CC=CC=C4
45	Verbthasin A	*Vt*	[8]	[H][C@@]12COC(=C)[C@]1([H])C[C@@H](O)C2=CCO
SESQUITERPENES				
46	Buddlindeterpene A	*Vt*	[8]	CC2=CCC[C@]1(C)O[C@@H]1CC(C)(C)C=CC2=O
47	Buddlindeterpene B	*Vt*	[8]	CC1=CCC(C)(C)C=CC(=O)C(C)=CCC1
48	Buddlindeterpene C	*Vt*	[8]	C=C[C@@]1(C)CCC2C(O)(C1=O)[C@H]4CC3[C@@](C)(C)CCC[C@]23CO4
Triterpene Saponines				
49	Thapsuine B	*Vt*, *Vl*	[3,4,9]	[H][C@@]49C=C[C@]23OC[C@@]1(CCC(C)(C)C[C@]12[H])CC[C@@]3(C)[C@]4(C)CC[C@]%10([H])[C@](C)(CO)C(O[C@H]8OC(COC6OC(C)C(OC5OC(CO)C(O)C(O)C5O)C(O)C6O)[C@@H](O)C(OC7CC(C)C(O)C(O)C7O)[C@H]8O)CC[C@]9%10C
50	Hydroxythapsuine B	*Vt*	[3,4,9]	[H][C@@]49C=C[C@]23OC[C@@]1(CCC(C)(O)C[C@]12[H])CC[C@@]3(C)[C@]4(C)CC[C@]%10([H])[C@](C)(CO)C(O[C@H]8OC(COC6OC(C)C(OC5OC(CO)C(O)C(O)C5O)C(O)C6O)[C@@H](O)C(OC7CC(C)C(O)C(O)C7O)[C@H]8O)CC[C@]9%10C
51	Saikogenin A	*Vt*	[3,4,9]	CC1(CCC2(C(CC3(C(=C2C1)C=CC4C3(CCC5C4(CCC(C5(C)CO)O)C)C)C)O)CO)C
52	Thapsuine A	*Vt*, *Vl*	[3,4,9]	CC1C(C(C(C(O1)OC2C(C(OC(C2O)OC3CCC4(C(C3(C)CO)CCC5(C4C=CC67C5(CCC8(C6CC(CC8)(C)C)CO7)C)C)C)COC9C(C(C(C(O9)C)OC1C(C(C(C(O1)CO)O)O)O)O)O)O)O)O)O
53	Hydroxythapsuine A	*Vt*	[3,4,9]	CC%10OC(OC9C(O)C(COC2OC(C)C(OC1OC(CO)C(O)C(O)C1O)C(O)C2O)OC(OC8CC[C@]7(C)[C@H]6C=C[C@]45OC[C@@]3(CCC(C)(O)C[C@H]34)CC[C@@]5(C)[C@]6(C)CC[C@H]7[C@]8(C)CO)C9O)C(O)C(O)C%10O
54	Ursolic acid	*Vt*, *Vl*	[8]	CC1CCC2(CCC3(C(=CCC4C3(CCC5C4(CCC(C5(C)C)O)C)C)C2C1C)C)C(=O)O
55	Veratric acid	*Vt*	[8]	COC1=C(C=C(C=C1)C(=O)O)OC
56	β-spinasterol	*Vt*	[8]	[H]C(=C([H])C(CC)C(C)C)[C@@H](C)[C@@]4([H])CC[C@@]3([H])C2=CC[C@@]1([H])C[C@@H](O)CC[C@]1(C)[C@@]2([H])CC[C@@]34C
57	Hydroxythapsuine	*Vt*	[9]	[H][C@@]49C=C[C@]23OC[C@@]1(CCC(C)(O)C[C@]12[H])CC[C@@]3(C)[C@]4(C)CC[C@]%10([H])[C@](C)(CO)C(O[C@H]8OC(COC6OC(C)C(OC5OC(CO)C(O)C(O)C5O)C(O)C6O)[C@@H](O)C(OC7CC(C)C(O)C(O)C7O)[C@H]8O)CC[C@]9%10C
58	3-O-fucopyranosyl saikogenin F	*Vt*	[9]	C[C@@H]7O[C@H](OC6CC[C@]5(C)C4C=C[C@]23OC[C@@]1(CC[C@](C)(C)CC12)[C@@H](O)C[C@@]3(C)[C@]4(C)CCC5[C@]6(C)O)[C@@H](O)[C@H](O)[C@@H]7O
Phenilpropanoid Glycosides				
59	Verbascoside (=acetoside)	*Vs*, *Vl*	[9,16]	CC1C(C(C(C(O1)OC2C(C(OC(C2OC(=O)C=CC3=CC(=C(C=C3)O)O)CO)OCCC4=CC(=C(C=C4)O)O)O)O)O)O
60	Poliumoside	*Vs*, *Vt*, *Vb*	[9]	CC1C(C(C(C(O1)OCC2C(C(C(C(O2)OCCC3=CC(=C(C=C3)O)O)O)OC4C(C(C(C(O4)C)O)O)O)OC(=O)C=CC5=CC(=C(C=C5)O)O)O)O)O
61	Forsythoside B	*Vt*, *Vl*	[9]	CC1C(C(C(C(O1)OC2C(C(OC(C2OC(=O)C=CC3=CC(=C(C=C3)O)O)COC4C(C(CO4)(CO)O)O)OCCC5=CC(=C(C=C5)O)O)O)O)O)O
62	Arenarioside	*Vt*	[9]	CC1C(C(C(C(O1)OC2C(C(OC(C2OC(=O)C=CC3=CC(=C(C=C3)O)O)COC4C(C(C(CO4)O)O)O)OCCC5=CC(=C(C=C5)O)O)O)O)O)O
63	Alyssonoside	*Vt*	[9]	CC1C(C(C(C(O1)OC2C(C(OC(C2OC(=O)/C=C/C3=CC(=C(C=C3)O)OC)COC4C(C(CO4)(CO)O)O)OCCC5=CC(=C(C=C5)O)O)O)O)O)O
64	Leucosceptoside B	*Vt*	[9]	[H][C@@]5(O[C@@H]3C[C@H](OCCc1ccc(OC)c(O)c1)O[C@H](CO[C@@H]2OC[C@](O)(CO)[C@H]2O)[C@H]3OC(=C)C=Cc4ccc(O)c(OC)c4)O[C@@H](C)[C@H](O)[C@@H](O)[C@H]5O
65	Cistanoside B	*Vt*	[9]	CC1C(C(C(C(O1)OC2C(C(OC(C2OC(=O)/C=C/C3=CC(=C(C=C3)O)OC)COC4C(C(C(C(O4)CO)O)O)O)OCCC5=CC(=C(C=C5)O)OC)O)O)O)O
Flavones				
66	Apigenin	*Vl*	[8]	C1=CC(=CC=C1C2=CC(=O)C3=C(C=C(C=C3O2)O)O)O
67	Apigenin-7-glucuronide	*Vl*	[8]	C1=CC(=CC=C1C2=CC(=O)C3=C(C=C(C=C3O2)OC4C(C(C(C(O4)C(=O)O)O)O)O)O)O
68	Luteolin	*Vt*, *Vl*	[9,15]	C1=CC(=C(C=C1C2=CC(=O)C3=C(C=C(C=C3O2)O)O)O)O
69	Luteolin-5-glucoside	*Vl*	[8]	C1=CC(=C(C=C1C2=CC(=O)C3=C(O2)C=C(C=C3OC4C(C(C(C(O4)CO)O)O)O)O)O)O
70	Luteolin-7-glucuronide	*Vl*	[8]	C1=CC(=C(C=C1C2=CC(=O)C3=C(C=C(C=C3O2)OC4C(C(C(C(O4)C(=O)O)O)O)O)O)O)O
71	7-methoxy-luteolin	*Vl*	[8]	COc3cc(O)c2c(=O)cc(c1ccc(O)c(O)c1)oc2c3
72	Cynaroside	*Vt*	[9]	C1=CC(=C(C=C1C2=CC(=O)C3=C(C=C(C=C3O2)OC4C(C(C(C(O4)CO)O)O)O)O)O)O
73	Apigetrin	*Vt*	[9]	C1=CC(=CC=C1C2=CC(=O)C3=C(C=C(C=C3O2)OC4C(C(C(C(O4)CO)O)O)O)O)O
74	4′,7-dihydroxyflavone-4′-rhamnoside	*Vt*	[9]	C[C@H]4O[C@H](Oc3ccc(c2cc(=O)c1ccc(O)cc1o2)cc3)[C@@H](O)[C@@H](O)[C@@H]4O
75	6-hydroxyluteolin-7-glucoside	*Vt*	[9]	C1=CC(=C(C=C1C2=CC(=O)C3=C(C(=C(C=C3O2)OC4C(C(C(C(O4)CO)O)O)O)O)O)O)O
Flavonols				
76	Quercetin	*Vt*, *Vl*	[9]	C1=CC(=C(C=C1C2=C(C(=O)C3=C(C=C(C=C3O2)O)O)O)O)O
77	Quercetin-7-glucuronide	*Vl*	[8]	C1=CC(=C(C=C1C2=C(C(=O)C3=C(C=C(C=C3O2)OC4C(C(C(C(O4)C(=O)O)O)O)O)O)O)O)O
78	3′-methylquercetin	*Vt*	[9]	COC1=C(C=CC(=C1)C2=C(C(=O)C3=C(C=C(C=C3O2)O)O)O)O
79	Kaempferol	*Vt*	[9]	C1=CC(=CC=C1C2=C(C(=O)C3=C(C=C(C=C3O2)O)O)O)O
80	Rutin	*Vt*	[9]	CC1C(C(C(C(O1)OCC2C(C(C(C(O2)OC3=C(OC4=CC(=CC(=C4C3=O)O)O)C5=CC(=C(C=C5)O)O)O)O)O)O)O)O
O-Metilated Flavones				
81	Acacetin	*Vl*	[9]	COC1=CC=C(C=C1)C2=CC(=O)C3=C(C=C(C=C3O2)O)O
82	Acacetin-7-O-α-d-glucoside	*Vt*	[8]	O=c3cc(c1ccc(O)c(O)c1)oc4cc(OC2OC(CO)C(O)C(O)C2O)cc(O)c34
83	Patuletin	*Vl*	[8,9]	COC1=C(C2=C(C=C1O)OC(=C(C2=O)O)C3=CC(=C(C=C3)O)O)O

## Appendix B

**Table A2:** Targets and metabolites of *Verbascum* spp. Probability calculated by SwissTargetPrediction (http://www.swisstargetprediction.ch/ accessed date 10 June 2020).

Target Class	Target	Uniprot ID	Metabolite	Probability	Chemical Group
Cytochrome P450	Cytochrome P450 19A1	P11511	Apigenin	1.00	Flavone
			Quercetin	1.00	Flavonol
	Cytochrome P450 1B1	Q16678	3′-methylquercetin	1.00	Flavonol
			Kaempferol	1.00	
			Quercetin	1.00	
			Acacetin	1.00	O-metilated Flavone
			Apigenin	1.00	Flavone
			Luteolin	1.00	
Enzyme	11-beta- hydroxysteroid dehydrogenase 1	P28845	Ursolic acid	0.66	Triterpene
	Aldehyde reductase (by homology)	P14550	Quercetin	0.66	Flavonol
	Aldo-keto reductase family 1 member B10	O60218	Apigenin	0.68	Flavone
			Luteolin	0.68	
			Ursolic acid	0.66	Triterpene
	Aldo-keto reductase family 1 member C1 (by homology)	Q04828	Quercetin	0.70	Flavonol
	Aldo-keto reductase family 1 member C2 (by homology)	P52895	Quercetin	0.73	Flavonol
	Aldo-keto reductase family 1 member C4 (by homology)	P17516	Quercetin	0.80	Flavonol
	Aldo-keto-reductase family 1 member C3 (by homology)	P42330	Quercetin	0.80	Flavonol
	Aldose reductase	P15121	Luteolin	1.00	Flavone
			Apigenin	1.00	
			Apigenin-7-glucuronide	1.00	
			7-methoxy-luteolin	1.00	
			Luteolin-7-glucuronide	1.00	
			Quercetin	1.00	Flavonol
			Kaempferol	1.00	
	Arachidonate 12-lipoxygenase	P18054	Quercetin	1.00	Flavonol
	Arachidonate 15-lipoxygenase	P16050	Quercetin	1.00	Flavonol
	Arginase-1 (by homology)	P05089	Luteolin	1.00	Flavone
	DNA polymerase beta	P06746	Ursolic acid	1.00	Triterpene
	DNA-(apurinic or apyrimidinic site) lyase	P27695	Quercetin	1.00	Flavonol
	Estradiol 17-beta-dehydrogenase 1	P14061	Apigenin	1.00	Flavone
			Luteolin	1.00	
			Kaempferol	1.00	Flavonol
	Estradiol 17-beta-dehydrogenase 2	P37059	Kaempferol	1.00	Flavonol
		P37059	Quercetin	1.00	Flavonol
	Glyoxalase I	Q04760	Luteolin	1.00	Flavone
			Kaempferol	1.00	Flavonol
			Quercetin	1.00	
	Liver glycogen phosphorylase	P06737	Quercetin	1.00	Flavonol
	Lymphocyte differentiation antigen CD38	P28907	Luteolin	1.00	Flavone
	Myeloperoxidase	P05164	Quercetin	1.00	Flavonol
	NADPH oxidase 4	Q9NPH5	Apigenin	1.00	Flavone
			Luteolin	1.00	
			Kaempferol	1.00	Flavonol
			Quercetin	1.00	
	Phospholipase A2 group B	P04054	Quercetin	1.00	Flavonol
	PI3-kinase p110-gamma subunit	P48736	Quercetin	1.00	Flavonol
	PI3-kinase p85-alpha subunit	P27986	Quercetin	1.00	Flavonol
	Poly [ADP-ribose] polymerase-1	P09874	Luteolin	1.00	Flavone
	Tankyrase-1	O95271	Apigenin	1.00	Flavone
			Luteolin	1.00	
	Tankyrase-2	Q9H2K2	Apigenin	1.00	Flavone
			Luteolin	1.00	
Eraser	Lysine-specific demethylase 4D-like	B2RXH2	Quercetin	0.68	Flavonol
Family A G protein- coupled receptor	Adrenergic receptor alpha-2	P18825	Rutin	1.00	Flavonol
	Alpha-2a adrenergic receptor	P08913	Rutin	1.00	Flavonol
	Neuromedin-U receptor 2	Q9GZQ4	Rutin	1.00	Flavonol
	Adenosine A1 receptor (by homology)	P30542	Luteolin	1.00	Flavone
			Apigenin	1.00	
			Kaempferol	0.80	Flavonol
			Quercetin	1.00	
	Adenosine A2a receptor (by homology)	P29274	Apigenin	1.00	Flavone
			Quercetin	1.00	Flavonol
	Dopamine D4 receptor	P21917	Quercetin	1.00	Flavonol
	G-protein coupled receptor 35	Q9HC97	Quercetin	1.00	Flavonol
	Interleukin-8 receptor A	P25024	Quercetin	1.00	Flavonol
	Vasopressin V2 receptor	P30518	Quercetin	1.00	Flavonol
Hydrolase	Acetylcholinesterase	P22303	Apigenin	1.00	Flavone
			Kaempferol	0.77	Flavonol
			Quercetin	0.68	
			Rutin	1.00	
Isomerase	DNA topoisomerase I (by homology)	P11387	Luteolin	1.00	Flavone
	DNA topoisomerase II alpha	P11388	Quercetin	0.68	Flavonol
Kinase	ALK tyrosine kinase receptor	Q9UM73	Quercetin	1.00	Flavonol
	CaM kinase II beta	Q13554	Quercetin	1.00	Flavonol
	Casein kinase II alpha	P68400	Apigenin	1.00	Flavone
			Quercetin	1.00	Flavonol
	Cyclin-dependent kinase 1	P06493	Quercetin	1.00	Flavonol
	Cyclin-dependent kinase 5/CDK5 activator 1	Q15078	Apigenin	1.00	Flavone
			Luteolin	1.00	Flavone
	Cyclin-dependent kinase 6	Q00534	Apigenin	1.00	Flavone
	Death-associated protein kinase 1	P53355	Quercetin	1.00	Flavonol
	Epidermal growth factor receptor erbB1	P00533	Quercetin	1.00	Flavonol
	Focal adhesion kinase 1	Q05397	Quercetin	1.00	Flavonol
	Glycogen synthase kinase-3 beta	P49841	Apigenin	1.00	Flavone
			Luteolin	1.00	
			Kaempferol	0.66	Flavonol
			Quercetin	1.00	
	Hepatocyte growth factor receptor	P08581	Quercetin	1.00	Flavonol
	Insulin receptor	P06213	Quercetin	0.68	Flavonol
	Insulin-like growth factor I receptor	P08069	Quercetin	1.00	Flavonol
	Myosin light chain kinase, smooth muscle	Q15746	Quercetin	0.68	Flavonol
	NUAK family SNF1-like kinase 1	O60285	Quercetin	1.00	Flavonol
	Protein kinase N1	Q16512	Quercetin	1.00	Flavonol
	Protein kinase C alpha	P17252	Verbascoside	0.78	Phenilpropanoid
	Serine/threonine-protein kinase AKT	P31749	Quercetin	1.00	Flavonol
	Serine/threonine-protein kinase Aurora-B	Q96GD4	Quercetin	1.00	Flavonol
	Serine/threonine-protein kinase NEK2	P51955	Quercetin	1.00	Flavonol
	Serine/threonine-protein kinase NEK6	Q9HC98	Quercetin	1.00	Flavonol
	Serine/threonine-protein kinase PIM1	P11309	Quercetin	1.00	Flavonol
	Serine/threonine-protein kinase PLK1	P53350	Quercetin	1.00	Flavonol
	Tyrosine-protein kinase receptor FLT3	P36888	Apigenin	1.00	Flavone
			Luteolin	1.00	
			Kaempferol	1.00	Flavonol
			Quercetin	1.00	
	Tyrosine-protein kinase receptor UFO	P30530	Quercetin	1.00	Flavonol
	Tyrosine-protein kinase SRC	P12931	Quercetin	1.00	Flavonol
	Tyrosine-protein kinase SYK	P43405	Apigenin	1.00	Flavone
			Luteolin	1.00	
			Kaempferol	0.66	Flavonol
			Quercetin	0.70	
	Vascular endothelial growth factor receptor2	P35968	Quercetin	1.00	Flavonol
Lyase	Carbonic anhydrase I	P00915	Quercetin	1.00	Flavonol
	Carbonic anhydrase II	P00918	Luteolin	1.00	Flavone
				1.00	
			3′-methylquercetin	1.00	Flavonol
			Kaempferol	1.00	
			Quercetin	1.00	
	Carbonic anhydrase III	P07451	Quercetin	1.00	Flavonol
	Carbonic anhydrase IV	P22748	Luteolin	1.00	Flavone
			3′-methylquercetin	1.00	Flavonol
			Kaempferol	1.00	
			Quercetin	1.00	
	Carbonic anhydrase IX	Q16790	Quercetin	1.00	Flavonol
	Carbonic anhydrase VA	P35218	Quercetin	1.00	Flavonol
	Carbonic anhydrase VI	P23280	Quercetin	1.00	Flavonol
	Carbonic anhydrase VII	P43166	Luteolin	1.00	Flavone
			Kaempferol	1.00	Flavonol
			Quercetin	1.00	
	Carbonic anhydrase XII	O43570	Luteolin	1.00	Flavone
			3′-methylquercetin	1.00	Flavonol
			Kaempferol	1.00	
			Quercetin	1.00	
	Carbonic anhydrase XIII (by homology)	Q8N1Q1	Quercetin	1.00	Flavonol
	Carbonic anhydrase XIV	Q9ULX7	Quercetin	1.00	Flavonol
	Carbonic anhydraseVII	P43166	3′-methylquercetin	1.00	Flavonol
Membrane receptor	Beta amyloid A4 protein	P05067	Luteolin	1.00	Flavone
	Receptor-type tyrosine-protein phosphatase F (LAR)	P10586	Ursolic acid	0.70	Triterpene
Nuclear receptor	Estrogen receptor alpha	P03372	Apigenin	1.00	Flavone
	Estrogen receptor beta	Q92731	Apigenin	1.00	Flavone
	Estrogen-related receptor alpha	P11474	Kaempferol	1.00	Flavonol
	Nuclear receptor ROR-gamma	P51449	Ursolic acid	0.70	Triterpene
Other cytosolic protein	Cyclin-dependent kinase 1/cyclin B	Q8WWL	Apigenin	1.00	Flavone
	Cyclin-dependent kinase 1/cyclin B	Q8WWL7	Luteolin	1.00	Flavone
	Heat shock protein HSP 90-alpha	P07900	Catalposide	0.83	Iridoid
			Specioside	1.00	
Other ion channel	Cystic fibrosis transmembrane conductance regulator	P13569	Apigenin	1.00	Flavone
Oxidoreductase	Arachidonate 5-lipoxygenase	P21397	Apigenin	1.00	Flavone
		P35354	Apigenin	1.00	
		P47989	Apigenin	1.00	
		P09917	Luteolin	1.00	
		P21397	Luteolin	1.00	
		P47989	Luteolin	1.00	
		P47989	3′-methylquercetin	1.00	Flavonol
		P09917	Kaempferol	1.00	
		P14679	Kaempferol	1.00	
		P21397	Kaempferol	0.66	
		P47989	Kaempferol	1.00	
		P09917	Quercetin	1.00	
		P21397	Quercetin	1.00	
		P47989	Quercetin	1.00	
Phosphatase	Low molecular weight phosphotyrosine protein phosphatase	P24666	Ursolic acid	0.7	Triterpene
	Protein-tyrosine phosphatase 1B	P18031	Ursolic acid	0.95	Triterpene
	Receptor-type tyrosine-protein phosphatase S	Q13332	Luteolin	0.90	Flavone
	T-cell protein-tyrosine phosphatase	P17706	Ursolic acid	0.74	Triterpene
Primary active transporter	ATP-binding cassette sub-family G member 2	Q9UNQ0	Luteolin	1.00	Flavone
			Kaempferol	1.00	Flavonol
			Quercetin	1.00	
			Apigenin	1.00	Flavone
	Multidrug resistance-associated protein 1	P33527	Apigenin	1.00	Flavone
			Luteolin	0.66	
			Kaempferol	1.00	Flavonol
			Quercetin	1.00	
	P-glycoprotein 1	P08183	Kaempferol	1.00	Flavonol
			Quercetin	1.00	
Protease	Beta-secretase 1	P56817	Quercetin	1.00	Flavonol
	Matrix metalloproteinase 12	P39900	Luteolin	1.00	Flavone
			Poliumoside	0.68	Phenilpropanoid
			Verbascoside	0.74	
	Matrix metalloproteinase 13	P45452	Quercetin	1.00	Flavonol
	Matrix metalloproteinase 2	P08253	Luteolin	1.00	Flavone
			Kaempferol	0.66	Flavonol
			Quercetin	1.00	
			Poliumoside	0.68	Phenilpropanoid
			Verbascoside	0.74	
	Matrix metalloproteinase 3	P08254	Quercetin	1.00	Flavonol
	Matrix metalloproteinase 9	P14780	Luteolin	1.00	Flavone
			Kaempferol	0.66	Flavonol
			Quercetin	1.00	
	Plasminogen	P00747	7-methoxy-luteolin	1.00	Flavone
	Thrombin	P00734	Quercetin	1.00	Flavonol
Secreted protein	Interleukin-2	P60568	6-hydroxyluteolin-7-glucoside	0.70	Flavone
			Apigetrin	0.66	
			Cynaroside	1.00	
			Acacetin-7-O-α-D-glucoside	1.00	O-metilated Flavone
	TNF-alpha	P01375	6-hydroxyluteolin-7-glucoside	0.70	Flavone
			Apigetrin	1.00	
			Cynaroside	1.00	
			Acacetin-7-O-α-D-glucoside	1.00	O-metilated Flavone
	Transthyretin	P02766	Apigenin	1.00	Flavone
			Luteolin	1.00	
Transcription factor	Aryl hydrocarbon receptor	P35869	Kaempferol	1.00	Flavonol
	Zinc finger protein GLI1	P08151	Buddlindeterpene B	0.74	Sesquiterpene
	Zinc finger protein GLI2	P10070	Buddlindeterpene B	0.74	Sesquiterpene
Unclassified protein	Microtubule-assicuated protein tau	P10636	Quercetin	0.68	Flavonol
